# Cognitive, behavioral and socio-communication skills as predictors of response to Early Start Denver Model: a prospective study in 32 young children with Autism Spectrum Disorder

**DOI:** 10.3389/fpsyt.2024.1358419

**Published:** 2024-05-30

**Authors:** Lisa Asta, Tiziana Di Bella, Francesca La Fauci Belponer, Marianna Bruschetta, Silvia Martines, Enrica Basile, Maria Boncoddo, Fabiana Bellomo, Francesca Cucinotta, Arianna Ricciardello, Laura Turriziani, Costanza Colombi, Federico Banchelli, Riccardo Cuoghi Costantini, Roberto D’Amico, Antonio M. Persico

**Affiliations:** ^1^ Department of Biomedical, Metabolic and Neural Sciences, University of Modena and Reggio Emilia, Modena, Italy; ^2^ Interdepartmental Program “Autism 0–90”, “G. Martino” University Hospital, Messina, Italy; ^3^ Institute for Biomedical Research and Innovation (IRIB), National Research Council of Italy (CNR), Messina, Italy; ^4^ IRCCS Centro Neurolesi “Bonino-Pulejo”, Messina, Italy; ^5^ Center for Autism “Dopo di noi”, Barcellona Pozzo di Gotto, Messina, Italy; ^6^ IRCCS Stella Maris Foundation, Pisa, Italy; ^7^ Department of Medical and Surgical Sciences, University of Modena and Reggio Emilia, Modena, Italy; ^8^ Unit of Statistical and Methodological Support to Clinical Research, Modena University Hospital, Modena, Italy; ^9^ Child and Adolescent Neuropsychiatry Program, Modena University Hospital, Modena, Italy

**Keywords:** autism, early intervention, Early Start Denver Model, ESDM, predictors, naturalistic developmental behavioral interventions, NDBI

## Abstract

**Introduction:**

The effectiveness of early interventions in young autistic children is well established, but there is great interindividual variability in treatment response. Predictors of response to naturalistic developmental behavioral interventions (NDBI), like the Early Start Denver Model (ESDM), are needed.

**Methods:**

We conducted an exploratory study to prospectively seek predictors of response in 32 young children treated with ESDM after receiving an ASD diagnosis. All children were less than 39 months old (mean age: 29.7 mo), and received individualized ESDM for nine months. Tests were administered at the beginning, after 4 months, and at the end of treatment.

**Results:**

Four children (12.5%) were “strong responders”, 8 children (25.0%) were “moderate responders”, and 20 children (62.5%) were “poor responders”. A more favorable response to ESDM was significantly predicted by higher PEP-3 Expressive Language, Receptive Language, Cognitive Verbal/Preverbal, Visuo-Motor Imitation scores, higher GMDS-ER Personal/Social, and VABS-II Communication scores, by lower ADI-R C restricted/stereotypic behaviors, and by joint attention level.

**Discussion:**

Most predictors showed a linear association with increasing response to ESDM, but GMDS-ER Personal-Social and joint attention level predicted strong response, while PEP-3 receptive language equally predicted moderate or strong response. Although larger samples will be necessary to reach definitive conclusions, in conjunction with prior reports our findings begin providing information able to assist clinicians in choosing the most appropriate treatment program for young autistic children.

## Introduction

Autism Spectrum Disorder (ASD) is a heterogeneous, neurodevelopmental condition present from early childhood, characterized by persistent deficits in social communication and interaction, repetitive behaviors, restricted interests or activities, and anomalous sensory processing (1). Currently, researchers emphasize the importance of early detection of autistic features in infants and pre-schoolers, in order to provide children with treatment and support as soon as possible (2–4). There is strong evidence showing that interventions implemented in early childhood, when brain development is most sensitive to early experiences, positively impact children’s developmental trajectories (5–18). Specifically, results from reviews and meta-analyses suggest that early interventions have positive effects on social communication, expressive and receptive language, cognitive skills and adaptive behaviors (19–26). Research has also shown that gains on several outcome measures, such as cognitive skills, language, adaptive behaviors and socio-communication abilities, are maintained years after treatment cessation (27–31).

Nevertheless, autism displays impressive heterogeneity, ranging from its genetic and epigenetic underpinnings up to its clinical manifestations, and also in the different neurocognitive mechanisms that appear to underlie the disorder (32, 33). Not surprisingly, this heterogeneity is reflected in intervention outcome, with some autistic children achieving remarkable results while others showing only little progress. Moreover, several different approaches to early intervention have been designed, and it can be very stressful and frustrating for families to choose the best intervention for their own child (34). For this reason, researchers are attempting to identify factors that may guide clinicians in prescribing the most effective intervention for each child (32, 35). Several child characteristics have been proposed to predict subsequent response to early intervention, such as younger age at treatment start (36–43) or better cognitive abilities at intake (5, 9, 10, 15, 36–38, 40, 44–50).

The Early Start Denver Model (ESDM) is a manualized, comprehensive early intervention designed for children with autism aged 12–48 months (51). The ESDM is part of the Naturalistic Developmental Behavioral Interventions (NDBI), which are interventions based on children’s learning that include naturalistic, developmental and behavioral components, emphasizing children’s spontaneous initiatives, rather than responses to prompts (52, 53). NDBI intervention types are considered among the most effective in improving autistic children development (54). The ESDM is a comprehensive approach stimulating skills across eight developmental domains, including receptive communication, expressive communication, social skills, play skills, cognitive skills, fine motor, gross motor, and adaptive behavior. Before treatment start, children’s abilities are assessed according to the ESDM Curriculum Checklist (51) to identify specific, short-term objectives to be learned in the following weeks. The curriculum checklist is readministered every 12 weeks until the end of treatment, to monitor children’s progress across all developmental domain. Several studies have proven the efficacy and effectiveness of the ESDM, with children receiving this intervention improving more than their autistic peers receiving treatment as usual or no treatment on overall developmental quotient (DQ), language and communication skills (12, 14, 55). Moreover, these gains persist after the end of treatment (30).

The present study was conducted on a sample of 32 young autistic children entering treatment and receiving individualized ESDM sessions for nine months before reaching the age of 48 months. Based on the evidence summarized above, we expected that group-wise our sample would collectively benefit from ESDM, especially in the language, socio-communication, and cognitive domains, but that at an individual level, children would display great variability in treatment response. Therefore, the purpose of this study was twofold: 1) to quantify the rate of Strong Responders (SR), Moderate Responders (MR), and Poor Responders (PR) to ESDM in our sample, categorized applying the set of criteria described below, and 2) to define the clinical and psychodiagnostic characteristics best able to predict strong, moderate or poor response prior to starting ESDM treatment.

## Methods

### Participants

Participants included in our sample were 26 (81.3%) boys and 6 (18.7%) girls, aged 20–39 months, referred for early intervention at the Interdepartmental Program “Autism 0–90” of the “G. Martino” University Hospital of Messina (Italy) after receiving an ASD diagnosis between 2016 and 2020. In addition to meeting DSM-5 diagnostic criteria for ASD (1) with complete agreement between at least two child neuropsychiatrists, children were included if their chronological age was between 20 and 39 months at treatment start, in order to ensure that the 9 month-long intervention would be entirely performed within the 20–48 month age range for which the ESDM was originally designed (51). All parents were Caucasians of Italian descent, except for one, and in all families Italian was the spoken language. Data on socioeconomic status and parents’ educational attainment levels were not recorded. Children were excluded in the presence of a neurodevelopmental disorder of known genetic aetiology (e.g., Fragile X Syndrome), neurological disorders (e.g., epilepsy) or focal neurological signs, or brain malformations at the MRI. The main clinical and psychodiagnostic pre-treatment characteristics of the sample are summarized in **Table 1**.

**Table 1:** Clinical and psychodiagnostic pre-treatment characteristics of participants.

	N	Mean (SD)	Range
**Age**	32	29.66 (4.48)	20–39
**Sex (M:F)**	32	26:6	—
**ADOS-2 Total Score**	24	17.83 (4.78)	9–30
**GMDS-ER Global Developmental Quotient**	32	60.8 (17.72)	21–100
**VABS-II Composite Score**	25	66.33 (12.07)	42–88

ADOS-2, Autism Diagnostic Observation Schedule – Second Edition; GMDS-ER, Griffiths Mental Developmental Scales – Extended Revised; VABS-II, Vineland Adaptive Behavior Scales – Second Edition.

### Procedures and measures

Following the clinical diagnosis of ASD, children underwent a comprehensive assessment and ESDM treatment was started within 3 months of referral. Based on the assessment of children’s skills conducted before treatment using the ESDM Curriculum Checklist (51), an individualized ESDM plan was developed for each child. Children received 6 hours per week of one-to-one intervention during four 90-minute sessions per week for a duration of nine months. Sessions were delivered by three ESDM-certified therapists per each child at the Interdepartmental Program “Autism 0–90” under the supervision of an ESDM Certified Trainer (Costanza Colombi). ESDM-certified therapists received their certification from the Trainer after at least two years of training and supervision. The coordinating therapist in charge of a single child delivered two sessions per week, while the other two therapists delivered one session each. One parent was present in the intervention room during all sessions. ESDM intervention was provided within the framework of the Italian National Health System; psychodiagnostic data collection and use was approved by the Ethical Committee of Messina (Italy) (June 19, 2017), and in accordance with the Helsinki Declaration, written informed consent for research use of the data was obtained from both parents of each child.

The psychodiagnostic measures analyzed for the present study include the Autism Diagnostic Observation Schedule – second edition (ADOS-2) (56), the Autism Diagnostic Interview-Revised (ADI-R) (57), the Griffiths Mental Developmental Scales-Extended Revised (GMDS-ER) (58), PsychoEducational Profile-Third Edition (PEP-3) (59), the Vineland Adaptive Behavior Scale – Second Edition (VABS-II) (60), and the Clinical Global Impression-Improvement (CGI-I) (61). All psychodiagnostic measures were administered immediately before starting ESDM (T0) and at the end of the 9-month treatment period (T2) by two psychologists (MB, FB) not involved in ESDM treatment and independent of the treatment team. A mid-term evaluation (T1) limited to the PEP-3 and to a set of visual analogue scales (VAS) was also carried out at four months, in order to monitor each child’s progress. At T0, 20 children were administered Module Toddler, 3 children Module 1 and one child Module 2. At T2, all children were readministered Module 1 except one, who received Module 2.

Finally, a set of early social skills, including eye contact, imitation, joint attention and play skills, were also qualitatively assessed during a semi-structured play session conducted during the intake visit by the child psychiatrist. Both child psychiatrists involved in this study (AMP, FC) achieved full inter-rater reliability on these measures, which were coded as follows:

- *Eye Contact*: normal, inconsistent, absent/very rare.
- *Imitation*: present, absent.
- *Play Skills*: pretend play, imitation play, manipulative/object play, disorganized play.
- *Joint Attention*:
*- Complete -* the child looks in the direction of pointing and then looks back and makes eye contact;
*- Incomplete* - the child looks in the direction of pointing, but does not look back and does not make eye contact.
*- Absent* - the child does not look in the direction of pointing.

These variables were not measured using structured scales but only this semi-structured classification, both to avoid making the psychodiagnostics assessment too cumbersome for the child, and because we preferred to evoke these functions using a consistent procedure and then to record response behaviors within the ecological context of a play session.

### Statistical analysis

A) *Group-wise treatment outcome analyses*: paired one-tailed *t*-tests were performed to compare pre- to post-intervention scores for ADOS-2, GMDS-ER, PEP-3 and VABS-II, under the assumption that children’s skills would improve, and not worsen, after nine months of ESDM. When dependent variables were not normally distributed, as indicated by Shapiro-Wilks test, paired one-tailed Wilcoxon tests were performed instead. Nominal p-values are reported.
B) *Case-wise treatment outcome analyses*: two expert child neuropsychiatrists, not directly involved in administering ESDM sessions, observed each child during a semi-structured play session at T0 and T2, independently providing CGI-I scores at T2 for each of the 32 children enrolled in this study. Few discrepancies were resolved reaching a final consensus. These consensus scores where then shared, explained and agreed upon with the entire équipe. The two primary domains used to define treatment outcome were “ASD severity” and “expressive language development”. Three response profiles were thus defined:
  1) *Strong Responders (SR)*: children who no longer meet DSM-5 criteria for ASD according to clinical evaluation *and* have acquired consistent verbal language (i.e., both words and sentences with typical fluency). At T2, these children receive a CGI-I score of 1 (“very much improved”), no longer exceed ADOS-2 diagnostic cut-offs for autism/autism spectrum, *and* display substantial improvements in the “Language” subscale of the GMDS-ER (i.e., ≥25%, unless the DQ was already aligned with the chronological age at T0, making this measure not informative due to a ceiling effect). In line with the general purpose of ESDM, i.e. to enhance cognitive, socioemotional, communication, motor and language skills in young autistic children (51), GMDS-ER scores were also expected to typically improve by >25% in several other subscales;
  2) *Moderate responders (MR)*, children who have largely improved in autism severity, but still meet DSM-5 criteria for ASD *and* have acquired some verbal language (i.e., single words or short sentences with limited fluency) or at least substantial non-verbal communication (i.e., pointing, hand waving, etc). At T2, these children receive a CGI-I score of 2 (“much improved”), in most cases display improved ADOS-2 total score by >25%, *and* GMDS-ER scores improved by >25% in 2–3 domains;
  3) *Poor responders (PR)*: children who still fully meet clinical DSM-5 criteria for ASD *and* have developed no or little verbal and non-verbal language. At T2, these children received a CGI-I score of 3 (“minimally improved”), ADOS-2 total scores either unchanged or improved by <25%, *and* GMDS-ER scores improved by >25% in few subscales, if any, with most subscale scores either unchanged, improving by <25%, or decreasing due to growing chronological age in the face of static skill acquisition.
The magnitude of change for each child between T0 and T2 was estimated as follows: [(T2-T0/T0*100)].
C) *Pre-treatment predictors of response*: this search was based on the previous Literature suggesting a possible association between response to ESDM and pre-treatment socio-communicative, language, and cognitive abilities (35). The following intake measures were thus selected: ADOS-2, ADI-R, GMDS-ER, PEP-3 and VABS-II subscales, joint attention, imitation, eye contact, play skills and chronological age. Single ordinal logistic regression (OLR) was employed for all metric independent variables, with “response to treatment” as dependent variable, and children’s intake measures as independent variables. Only in the case of the GMDS-ER Personal Social subscale score, the assumption that the odds ratio be the same across categories was violated (62) and a Multinomial Logistic Regressions was performed instead. When the predictor was categorical, Fisher’s exact test was employed. Nominal p-values are reported for quantitative variables, on the one hand, because no difference survives controls for multiple testing due to statistical power limitations imposed by small sample size and consequently to difficulties in reducing the number of variables based on inter-correlations (see below); on the other hand, considering the exploratory nature of this study. Bonferroni correction was instead applied to categorical variables. A principal component analysis (PCA) on variables that were significant predictors of response in single OLR was also conducted, in order to verify whether the number of variables explaining the variance in clinical outcome could be reduced.

All statistical analyses were performed in R, version 4.2.0 (63).

## Results

### Group-wise treatment outcome after ESDM

**Figure 1:**
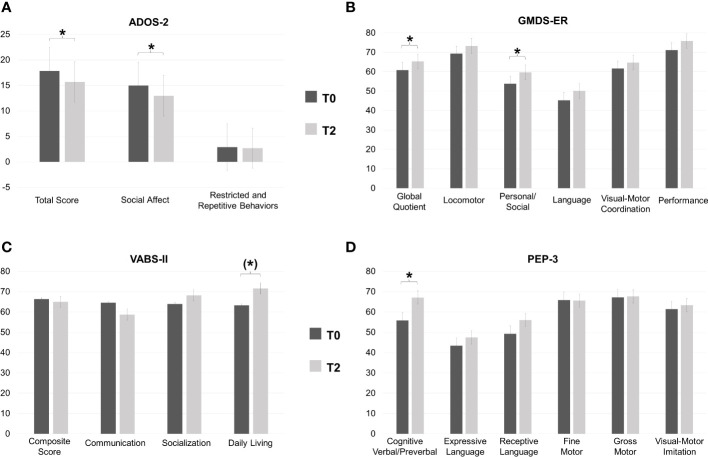
Group-wise mean (± S.D.) scores at baseline (T0) and post-treatment (T2) for: **(A)** Autism Diagnostic Observation Schedule – 2^nd^ (ADOS-2), **(B)** Griffith Mental Developmental Scales-Extended Revised (GMDS-ER), **(C)** PsychoEducational Profile – 3 (PEP-3), and **(D)** Vineland Adaptive Behavior Scale-II (VABS-II). *P<0.05, (*)P=0.05.

Collectively, the 32 young children who received a first diagnosis of ASD displayed some improvement with ESDM treatment, reaching nominal significance in several measures (**Figure 1**; **Supplementary Table S1**). In particular, a significant decrease between T0 and T2 was recorded in ADOS-2 SA (T0: 14.96; T2: 12.96; *p* = 0.01) and ADOS-2 Total score (T0: 17.83; T2: 15.67; *p* = 0.02), but not in the ADOS-2 RRB (T0: 2.88; T2: 2.71; *p* = 0.40), indicating that the improvement is specific to the social-affect domain. Children also improved significantly from T0 to T2 in the cognition domain, as indicated both by the GMDS-ER GQ (T0: 60.8; T2: 65.29; *p* = 0.04) and by the PEP-3 CVP (T0: 55.84; T2: 67.11; *p* = 0.02) subscales. Gains in VABS-II Daily Living approached statistical significance (T0: 63.25; T2: 71.6; *p* = 0.05). No other nominally significant difference between pre- and post-treatment was found in the remaining subscales (**Figure 1**; **Supplementary Table S1**).

### Case-wise treatment outcome analyses

**Figure 2:**
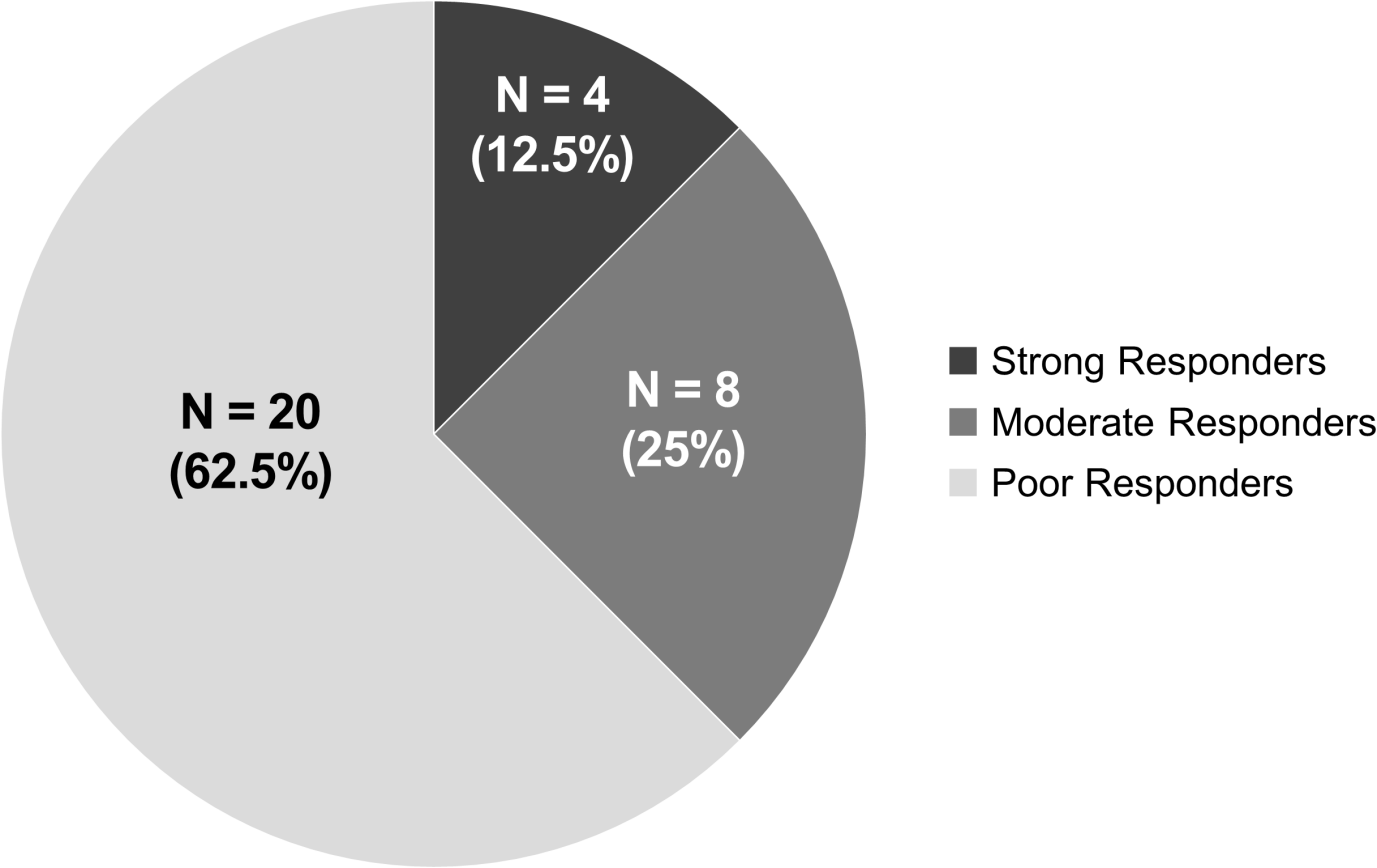
Proportions of strong, moderate, and poor responders to ESDM (n = 32).

Great interindividual variability in the magnitude of response to ESDM was observed. The overall treatment response profiles based on CGI-I are displayed in **Figure 2** and include 4 (12.5%) Strong Responders, 8 (25.0%) Moderate Responders, and 20 (62.5%) Poor Responders. Response profiles according to GMDS-ER and ADOS-2 subscale and total scores for each participant are presented in **Supplementary Table S2**. Noticeably, in addition to reduced autism severity and increased verbal language abilities, as detailed in the Methods section, Strong and Moderate Responders tend to display large (> 25%) improvements in several GMDS-ER subscales, indicating the efficacy of ESDM in stimulating a broad array of neurodevelopmental functions (**Supplementary Table S2**).

### Predictors of response to ESDM

**Figure 3:**
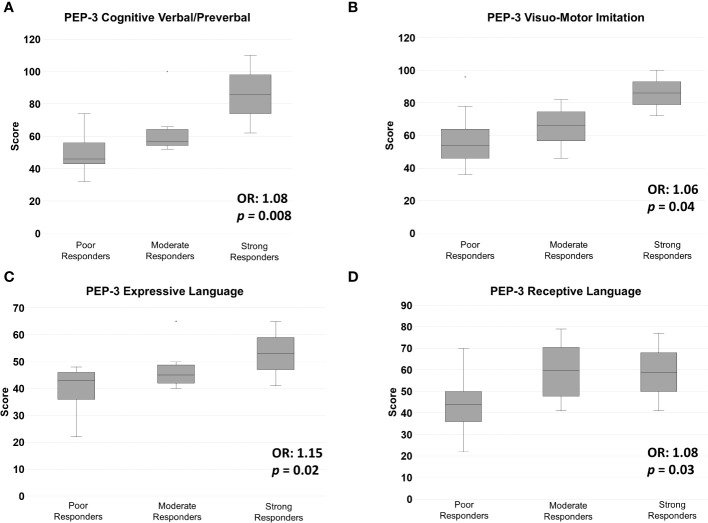
Pre-treatment mean (± S.D.) scores for the PEP-3 subscales: **(A)** Cognitive Verbal/Preverbal (CVP), **(B)** Visual Motor Imitation (VMI), **(C)** Expressive Language (EL), and **(D)** Receptive Language (RL), in Strong, Moderate and Poor Responders to ESDM. OR, odds ratio.

**Figure 4:**
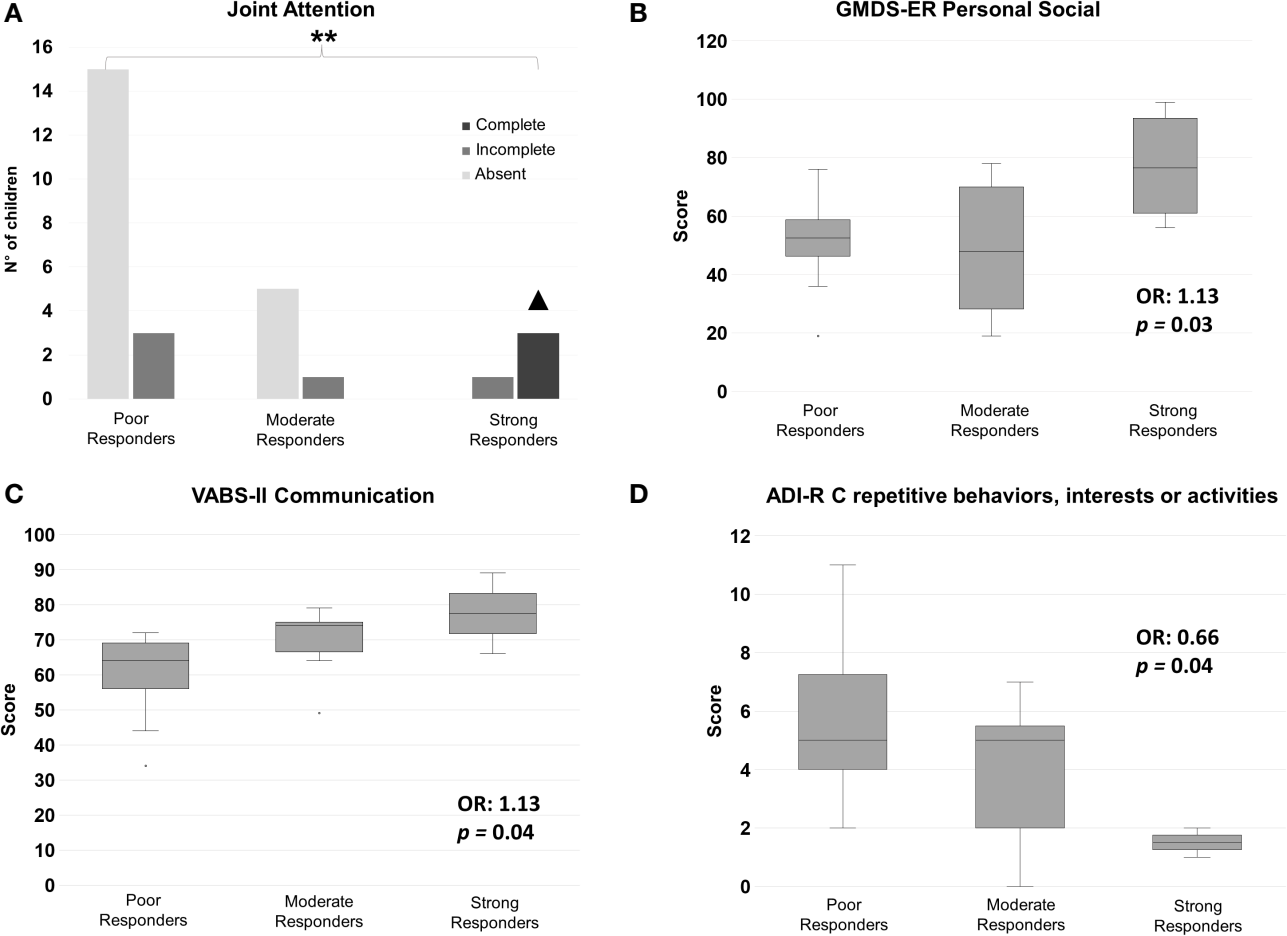
Pre-treatment **(A)** joint attention level, and mean (± S.D.) scores for **(B)** GMDS-ER Personal-Social subscale, **(C)** VABS-II Communication subscale, and **(D)** ADI-R C subscale – Repetitive, Restricted and Stereotyped Patterns of Behavior. OR: odds ratio. ** overall P<0.01. ▲ pairwise Strong Responders vs Poor Responders P<0.05.

Pre-treatment ADOS-2, ADI-R, GMDS-ER, PEP-3 and VABS-II subscale scores, as well as joint attention, imitation, eye contact, play skills and chronological age at T0, were analyzed to assess their predictive power over treatment response at T2. Significant results are listed in **Table 2** and displayed in **Figures 3**, **4**. Ordinal logistic regressions showed that the following measures collected at baseline were significantly associated with a more favorable ESDM outcome at T2: *a)* higher cognitive abilities, as measured with PEP-3 Cognitive Verbal/Preverbal score (*p* = 0.008) (**Figure 3A**); *b)* higher social skills (GMDS-ER Personal-Social scale: *p =* 0.03, **Figure 4B**); *c)* higher expressive and receptive language abilities measured with PEP-3 EL (*p* = 0.02) and RL (*p* = 0.03) scores, as displayed in **Figures 3C, D**, respectively; *d)* higher communication skills (VABS-II Communication; *p* = 0.04, **Figure 4C**); *e)* better visuo-motor imitation (PEP-3 VMI; *p* = 0.04, **Figure 3B**); *f)* less restricted and repetitive behaviors, interests or activities (ADI-R C: *p* = 0.04, **Figure 4D**). Also joint attention at intake was strongly associated with ESDM outcome (*p* = 0.002; **Figure 4A**). Most of these pre-treatment variables appear linearly distributed among strong, moderate and poor responders at T0 (**Figures 3**, **4**). The only exceptions were represented by PEP-3 receptive language scores, which were equally elevated in strong and moderate responders, compared to poor responders (**Figure 3D**); GMDS-ER Personal-Social scores, which were significantly elevated only in strong responders compared to poor responders (*p* = 0.03; **Figure 4B**); and complete joint attention, which is present at T0 only in strong responders (strong vs poor responders: pairwise *p*=0.01 after Bonferroni correction; **Figure 4A**). The remaining subscale scores did not reach statistical significance (**Supplementary Table S3**).

**Table 2:** Significant predictors of response to ESDM treatment: estimates, coefficients, statistics and p-values of Fisher’s Exact Test and logistic regression analyses.

							*Overall goodness of fit*	
	*N*	*b*	SE B	*z*	*p*	OR (CI)	*χ²*	*p*
**1. Joint Attention**	28	—	—	—	**0.002^†^**	—	—	—
**2. PEP-3 Cognitive Verbal/Preverbal**	25	0.08	0.03	2.65	**0.008**	1.08 (1.03–1.15)	8.99	0.003
**3. GMDS-ER Personal-Social**	32	0.13	0.06	2.14	**0.03**	1.13 (1.01–1.27)	9.80	0.007
**4. VABS-II Communication**	24	0.12	0.05	2.09	**0.04**	1.13 (1.02–1.29)	6.52	0.010
**5. PEP-3 Expressive Language**	25	0.14	0.06	2.24	**0.02**	1.15 (1.03–1.31)	6.17	0.012
**6. PEP-3 Receptive Language**	25	0.08	0.03	2.24	**0.03**	1.08 (1.01–1.16)	5.87	0.015
**7. ADI-R C Total Score**	25	-0.42	0.2	-2.08	**0.04**	0.66 (0.42–0.94)	5.66	0.02
**8. PEP-3 Visuo-Motor Imitation**	25	0.06	0.03	2.04	**0.04**	1.06 (1.00–1.13)	4.75	0.029

ADI-R, Autism Diagnostic Interview-Revised; CI, Confidence Interval; GMDS-ER, Griffith Mental Developmental Scale – Extended Revised; OR, Odd Ratio; PEP, Psychoeducational Profile; SE, Standard Error.

**^†^** Fisher’s exact test.

**Figure 5:**
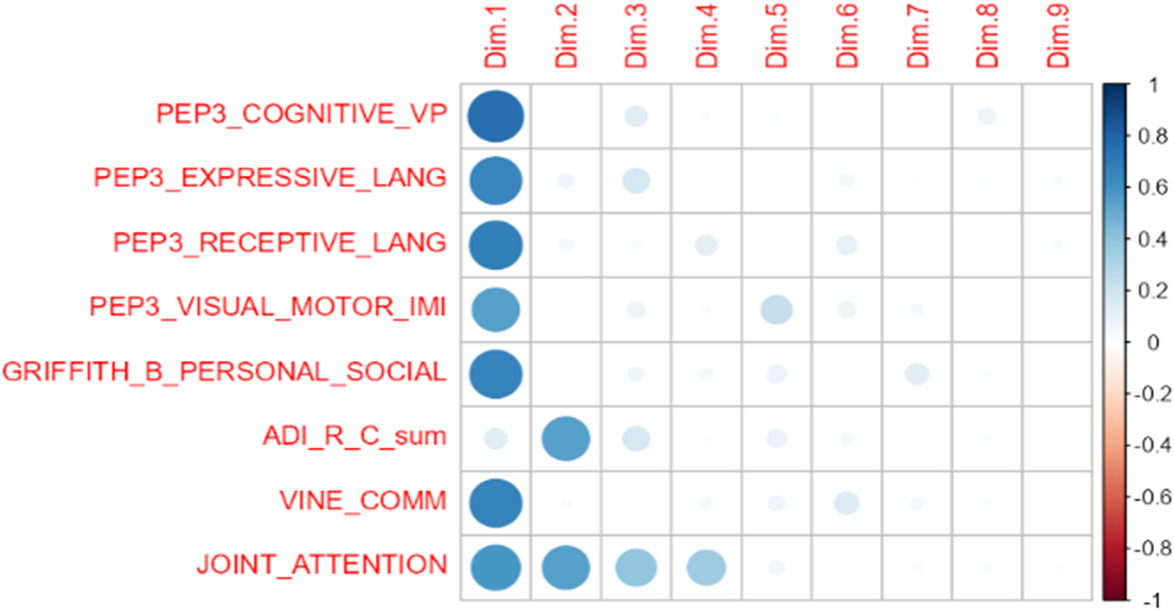
Graphical representation of principal component analysis (PCA). Dimension 1 was statistically significant (*p* = 0.01).

A principal component analysis (PCA) was then performed to verify whether we could reduce the number of predictors selecting one per principal component, and in order to estimate the variance in outcome explained by our set of significant predictors. The PCA identified a single significant Component I (0.8; CI: 0.16–1.42; *p* = 0.01), encompassing all significant pre-treatment predictors, except for ADI-R repetitive behaviors (**Figure 5**). This principal component explains 52.2% of the variance in post-treatment outcome.

## Discussion

The aims of this study were to define response rates to nine months of individualized ESDM treatment in an Italian sample of young children newly diagnosed with ASD, and to investigate baseline characteristics associated with a more favorable outcome. At a group level, participants significantly improved in DQ and overall cognitive abilities, personal-social skills, and core autistic symptoms (**Figure 1**; **Supplementary Table S1**). Interestingly, ADOS-2 SA and Total Score significantly decreased from baseline (T0) to post-intervention (T2) (**Supplementary Table S1**). This is noteworthy, since studies have not often found a statistically significant decrease in ADOS scores, even in the face of clinical improvement in symptom severity (41–43). Children also displayed some improvement in adaptive behaviors, as reflected by an increase in VABS-II Daily Living scores reaching a nominal p-value of 0.05 at T2. A global stimulation of the developmental trajectory was documented by the positive GMDS-ER percent differences recorded in many children (**Supplementary Table S2**). In the absence of a comparison group, we cannot rule out that these children would have improved regardless of the treatment they received. Nonetheless, the internal consistency of our results and their coherence with previous studies, showing that ESDM is effective in improving the developmental trajectory of young autistic children (e.g., 12, 15, 42), even if administered in a less intensive way than originally prescribed (i.e., 6 hr per week, e.g., 14, 41, 64), enhance confidence in their reliability. Great interindividual variability in children response to ESDM was observed, as expected (**Figure 2**). Based on CGI-I scores established by two expert clinicians, and then shared with the entire team, four (12.5%) children were categorized as Strong Responders, since at T2: *a*) they no longer fulfilled DSM-5 criteria for an ASD diagnosis, *b*) they did not exceed the ADOS-2 diagnostic threshold for autism, or autism spectrum, and *c*) they achieved impressive improvement in expressive language, as supported by scores obtained in several scales (**Supplementary Table S2**). Eight children (25.0%) acquired at least some verbal language and achieved sizable improvements in ADOS-2 and in several GMDS-ER subscale scores, but remained in the autism spectrum and were thus classified as Moderate Responders (MR). Finally, 20 children (62.5%) were categorized as Poor Responders (PR), because they achieved little clinical gain both in DSM-5 ASD severity and in language skills, although sizable improvement in ADOS-2 or in one or two GMDS domains was observed in several cases (**Supplementary Table S2**). The percentage of children who did not respond optimally to ESDM in our sample is higher than that reported in other studies (e.g., 49, 64, 65). This does not imply that our intervention did not have a positive effect on these children, since at group-level a statistically significant improvement was recorded in many domains. More likely, the discrepancy with previous results is related to the strict criteria used here to categorize children’s response to treatment, namely >25% improvement on multiple scales. Importantly, no child received a CGI-I score of 4 (“unchanged”), because we did observe some positive effects in each child, albeit to a different extent. This observation coincides with the anecdotal report provided by one therapist (EB) subsequently applying a more structured protocol based on Applied Behavioral Analysis (ABA) on seven of these twenty PR children, witnessing better child-therapist interactions and greater adjustment to the therapeutic setting compared to children of similar age and ASD severity starting behavioral treatment without prior ESDM. After determining the response level for each child, our main aim was to identify pre-treatment factors that would help predict a favorable response. Putative predictors were chosen among variables already found to be significantly related to ESDM outcome (35), namely social skills, expressive and receptive language, communication abilities and cognitive skills. We also investigated factors with weaker evidence in the Literature, such as age at treatment start, autistic symptom severity and adaptive behaviors, as well as early social skills including eye contact, joint attention, imitation and play. The latter skills are pivotal for learning, and the ESDM is specifically developed to boost joint attention and imitation as the “social infrastructure” for the acquisition of new skills (66). For this reason, despite mixed and insufficient evidence from previous studies, we hypothesized that children at a more advanced stage in the development of early social skills may be more likely to respond positively to ESDM. Among these early social skills, joint attention was most consistently associated to strong response to ESDM (**Figure 4**), in accordance with our hypothesis. In fact, all three children who displayed complete joint attention at intake were categorized as SR at post-treatment, while 15/20 (75.0%) of the children who completely lacked joint attention at baseline were classified as PR at the end of ESDM, with the remaining 5/20 (25.0%) displaying a moderate response. Surprisingly, yet in line with the previous Literature (e.g., 15, 64), eye contact, imitation and play reach marginal trends but not statistical significance (see **Supplementary Table S3**). This result seemingly places joint attention in a more pivotal position, compared to the other early social skills, pointing toward its possible role either as a functional “driver” of social development, or as an “early behavioral marker” (i.e., a behavioral response which requires an underlying neural network that at least partly must be in place to support all the other early social functions).

Our results confirmed the importance of greater communication and language skills at intake, as measured by VABS-II Communication, PEP-3 EL and PEP-3 RL, in predicting a positive response to intervention (42, 48, 50, 64). Interestingly, on the one hand VABS-II Communication and PEP-3 EL pre-treatment scores follow a linear upward trend in the three response groups, with SR scoring the highest, PR the lowest, and MR somewhere in between (**Figures 3C**, **4C**, respectively). On the other hand, strong and moderate responders obtained overlapping scores on PEP-3 Receptive Language (**Figure 3D**). These data suggest that starting treatment with at least some receptive language may be a pre-requisite to achieve a satisfactory response to ESDM, while better communication skills and some expressive language may be required for strong response.

Baseline verbal/preverbal cognitive abilities were also found to predict a better response to ESDM: children with higher PEP-3 CVP scores at T0 were also more likely to be strong responders (**Figure 3A**). However, no significant association was found with overall DQ. Higher DQ at intake has often been reported as a major predictor of positive response to behavioral intervention (e.g., 5, 11, 15, 46, 48, 49, 64, 67); nevertheless, not all Authors have found such association especially in ESDM studies (for review see 35).

Strong response was also associated with greater visuo-motor imitation abilities at intake, as measured by PEP-3 VMI. To our knowledge, this factor has been investigated as predictor of treatment response only in one other study, which found it to be associated with early intervention outcome (68). Also lower lifetime levels of repetitive and stereotyped behaviors, as recorded from caregivers by the ADI-R subscale C score, are associated with better ESDM response here (**Table 2**; **Figure 4D**), in line with two other recent studies (17, 65). Finally, higher adaptive behaviors, as measured using the VABS-II, have been more frequently observed post-treatment as a result of successful ESDM intervention (6, 44, 68, 69), than pre-treatment as a predictor of subsequent response (46, 47). Our study confirms this trend, conceivably due to adaptive behaviors representing a complex multifunctional construct, well fit to represent a global outcome measure, but not sufficiently analytical to define the specific factors contributing to treatment response. Furthermore, the VABS-II collects parental reports and this adds an additional layer of complexity, as compared to scales based on the direct observation of the child by clinicians or therapists, such as the PEP-3.

Younger age at treatment start did not affect response to ESDM intervention in our sample (*b* = 0.08; *p* = 0.32). This finding may seem counterintuitive, as several studies have documented that younger children seem to respond better to early intervention (e.g., 36, 37, 39, 41–43). However, this could be explained by the very narrow age range of the children enrolled in this study. In fact, similar studies including only children under 48 months at intake have not found age at intake to be associated with treatment response (e.g., 5, 47, 64, 68).

This study presents at least three limitations, which must be duly acknowledged. First, the overall sample size is relatively small and the three response groups highly unbalanced. There are fewer strong and moderate responders compared to poor responders, and this may affect our results. Conceivably, to minimize the impact of this issue and to simplify the study design, strong and moderate responders could be merged into one single “Responders” group and compared with poor responders. However, we decided not to proceed in this way, because strong and moderate responders clinically differ in many areas, including prognosis, developmental trajectory, clinical management, needs, type of future interventions, etc. This is indirectly supported also by the relative of our results in terms of relatively narrow standard deviations (**Figures 3**, **4**). In contrast to moderate responders, who achieved significant gains but still remained in the autism spectrum, strong responders improved to such an extent that after nine months of treatment they no longer met DSM-5 diagnostic criteria for ASD and ADOS-2 criteria for autism/autism spectrum, while achieving a broad-based improvement in most or all developmental domains (**Supplementary Table S2**). We cannot exclude that later in life some autistic features may again emerge and require updating the clinical diagnosis, nor do we deny that children who leave the autism spectrum still do require to be followed up for the frequent occurrence of other neurodevelopmental issues (70). Nonetheless, distinguishing three levels of response better fits the clinical reality of autism. Our original design has thus been maintained, although sample size limitations must be considered and the present results should be cautiously interpreted within the broader framework of the existing Literature (35).

Secondly, we investigated the predictive power of measures, including ADOS-2 and GMDS-ER, that were also related to the outcome of interest. In fact, the three response categories (i.e., strong, moderate, and poor responders) were primarily based on the CGI-I, but also ADOS-2 and GMDS-ER served an ancillary role in outcome determination (**Supplementary Table S2**). At first glance, it may appear inappropriate that the same measure be used to define outcome and to predict outcome. However, it must be noted that as outcome measure we used the magnitude of pre-/post-treatment change (**Supplementary Table S2**), whereas predictive power was explored by regression models using the T0 raw scores of these measures as independent variables. This strategy thus prevents the tautological invalidation of these analyses, which retain their full validity. Furthermore, our treatment response criteria may appear to be placing too much emphasis on autistic behaviors, whereas ESDM was designed to enhance cognitive, socioemotional, communication, motor and language skills in young autistic children (51). This broader scope was indeed considered in our study, by measuring all major developmental domains using the GMDS-ER (**Supplementary Table S2**). However, in our real-life public clinical setting, children are prescribed an early intensive intervention because they receive a first diagnosis of ASD. The primary request from clinicians and families is understandably to ameliorate the behavioral features that justified this prescription. Hence in our context it is appropriate to maintain focus also on autistic behaviors, and not only on the broader developmental scenario, when defining response to treatment.

Thirdly, several predictors are significantly inter-correlated (**Supplementary Table S4**), as recognized also by PCA which groups almost all our significant predictors into a single component (**Figure 5**). Due to our limited sample size and to missing values, we were not able to reduce the number of variables by performing stepwise regressions. This in turn did not allow us to efficiently control for multiple comparisons without losing all statistical significance, an obvious type II error, as suggested by the consistency of our results with those of many prior studies. At the same time, reporting nominal p-values can indeed lead to an inflation of type I error and consequently to an overestimation of our findings.

Finally, although it does not represent a limitation *per se*, it should be emphasized that our results define a set of predictors of a favorable developmental trajectory during an ESDM intervention. Our study is not a randomized controlled trial (RCT) comparing ESDM to community treatment or to treatment as usual (e.g., 15), nor contrasting two well-defined early interventions targeting the outcome function of interest (e.g., EIBI, ESDM, JASPER, etc.) (e.g., 16). Hence, although children in our sample made significant gains groupwise especially in core autistic symptoms, cognitive, communication and early social domains, our experimental design only allows the identification of factors that contribute to a positive developmental trajectory during ESDM treatment, not treatment-related factors that predict children’s response to a specific intervention. In addition, improvements reported here cannot be ascribed to ESDM with absolute certainty, as they could also stem from a spontaneous developmental trajectory. Nevertheless, the design of our study is comparable to most research performed to date on this topic, namely single-group, pretest-posttest design (e.g., 48, 50, 64, 65, 71). Our results are comparable with those reported in these studies, and consistent with those reported by studies conducted in a more controlled fashion. A recent RCT comparing ESDM vs. CT (15) found that positive ESDM outcome was predicted by higher cognitive skills at baseline. Another study (18) comparing ESDM vs. EIBI found that higher DQ post-treatment was best predicted by joint attention, sustained attention and imitation at intake for both interventions, and that sustained attention in particular was a preferential predictor of ESDM response.

In conclusion, despite these limitations and caveats, the present results contribute some additional clinically useful information, to begin personalizing treatment in very young children newly diagnosed with ASD, within the broader context of an emerging field in autism research, striving to define “which treatment works best for which child”.

## Data availability statement

The datasets presented in this article will be made available by the corresponding author, upon reasonable request. Requests to access the datasets should be directed to antonio.persico@unimore.it.

## Ethics statement

The studies involving humans were approved by Ethics Committee of the University of Messina and of the “G. Martino” University Hospital, Messina, Italy (prot. n. 22/17, approved on June 19, 2017). The studies were conducted in accordance with the local legislation and institutional requirements. Written informed consent for participation in this study was provided by the participants’ legal guardians/next of kin.

## Author contributions

LA: Conceptualization, Validation, Writing – original draft. TD: Conceptualization, Data curation, Investigation, Validation, Writing – review & editing. FL: Investigation, Validation, Writing – review & editing. MBr: Investigation, Writing – review & editing. SM: Investigation, Writing – review & editing. EB: Investigation, Writing – review & editing. MBo: Investigation, Methodology, Writing – review & editing. FBe: Investigation, Writing – review & editing. FC: Data curation, Investigation, Writing – review & editing. AR: Data curation, Investigation, Validation, Writing – review & editing. LT: Data curation, Investigation, Validation, Writing – review & editing. CC: Conceptualization, Methodology, Supervision, Writing – review & editing. FBa: Formal analysis, Methodology, Writing – review & editing. RC: Formal analysis, Methodology, Writing – review & editing. RD’A: Conceptualization, Formal analysis, Methodology, Writing – review & editing. AP: Conceptualization, Funding acquisition, Methodology, Supervision, Validation, Writing – review & editing, Project administration.
