# Supplementary material for: Cognitive, behavioral and socio-communication skills as predictors of response to Early Start Denver Model: a prospective study in 32 young children with Autism Spectrum Disorder
Source: Front Psychiatry. 2024 May 30;15:1358419. doi: 10.3389/fpsyt.2024.1358419 (PMC11170145; doi:10.3389/fpsyt.2024.1358419)
Supplement: Supplementary file 1 [file Table_1.docx]

**Supplementary Material**

**Cognitive, behavioral and socio-communication skills as predictors of response to Early Start Denver Model:**

**A prospective study in 32 young children with Autism Spectrum Disorder.**

**Lisa Asta^1^, Tiziana Di Bella^2^, Francesca La Fauci Belponer^2^, Marianna Bruschetta^2^, Silvia Martines^2^, Enrica Basile^2^, Maria Boncoddo^3^, Fabiana Bellomo^2^, Francesca Cucinotta^4^, Arianna Ricciardello^2^, Laura Turriziani^5^, Costanza Colombi^6^, Federico Banchelli^7^, Riccardo Cuoghi Costantini^7^, Roberto D'Amico^7^, Antonio M. Persico^1,9^*.**

^1^ Department of Biomedical, Metabolic and Neural Sciences, University of Modena and Reggio Emilia, Modena, Italy,

^2^ Interdepartmental Program “Autism 0–90”, “G. Martino” University Hospital, Messina, Italy,

^3^ Institute for Biomedical Research and Innovation (IRIB), National Research Council of Italy (CNR), Messina, Italy,

^4^ IRCCS Centro Neurolesi "Bonino-Pulejo", Via Provinciale Palermo, Contrada Casazza, 98124 Messina, Italy

^5^ Center for Autism “Dopo di noi”, Barcellona Pozzo di Gotto, Messina, Italy,

^6^ IRCSS Stella Maris Foundation, Pisa, Italy,

^7^ Department of Medical and Surgical Sciences, University of Modena and Reggio Emilia, Modena, Italy,

^8^ Unit of Statistical and Methodological Support to Clinical Research, Modena University Hospital, Modena, Italy

^9^ Child and Adolescent Neuropsychiatry Program, Modena University Hospital, Modena, Italy

**Supplementary Table S1**. Mean (SD) pre-treatment (T0) and post-treatment (T2) scores. N=number of valid observations. Nominal one-tail p-values < 0.05 are highlighted in gray.

|  |  | **Pre-treatment (T0)** | |  | **Post-treatment (T2)** | | **Nominal**  ***p*-values** |
| --- | --- | --- | --- | --- | --- | --- | --- |
|  | ***N*** | **Mean (SD)** | **Range** | ***N*** | **Mean (SD)** | **Range** |  |
| **ADOS-2 Total score** | 24 | 17.83 (4.78) | 9-30 | 24 | 15.67 (5.12) | 6-23 | 0.02 |
| **ADOS-2 SA** | 24 | 14.96 (3.75) | 8-20 | 24 | 12.96 (4.71) | 3-19 | 0.01 |
| **ADOS-2 RRB** | 24 | 2.88 (2.19) | 0-10 | 24 | 2.71 (1.27) | 0-5 | 0.36 |
| **GMDS-ER GQ** | 32 | 60.8 (17.73) | 21-100 | 32 | 65.29 (17.89) | 43-111 | 0.04 |
| **GMDS-ER Locomotor** | 32 | 69.31 (21.13) | 24-124 | 32 | 73.25 (17.08) | 20-105 | 0.88 |
| **GMDS-ER Personal/Social** | 32 | 53.83 (17.22) | 19-99 | 32 | 59.69 (21.15) | 15-109 | 0.02 |
| **GMDS-ER Language** | 32 | 45.34 (18.26) | 14-103 | 32 | 50.15 (27.00) | 11-136 | 0.13 |
| **GMDS-ER Visuomotor Coordination** | 32 | 61.63 (20.11) | 17-103 | 32 | 64.75 (19.44) | 16-110 | 0.15 |
| **GMDS-ER Performance** | 31 | 71.09 (25.69) | 26-146 | 32 | 75.81 (26.01) | 17-125 | 0.19 |
| **PEP3 CVP** | 25 | 55.84 (18.37) | 32-110 | 24 | 67.11 (22.38) | 40-123 | 0.02 |
| **PEP-3 EL** | 25 | 43.36 (8.78) | 22-65 | 24 | 47.44 (18.98) | 25-83 | 0.20 |
| **PEP-3 RL** | 25 | 49.28 (14.57) | 22-79 | 24 | 56.01 (24.47) | 23-120 | 0.14 |
| **PEP-3 FM** | 25 | 65.92 (17.14) | 44-100 | 24 | 65.61 (11.35) | 44-88 | 0.70 |
| **PEP-3 GM** | 25 | 67.2 (17.16) | 44-103 | 24 | 67.64 (16.25) | 25-90 | 0.63 |
| **PEP-3 VMI** | 25 | 61.4 (17.38) | 36-100 | 24 | 63.36 (15.02) | 38-93 | 0.53 |
| **VABS-II CS** | 24 | 66.33 (12.07) | 42-88 | 23 | 65.00 (13.8) | 31-92 | 0.63 |
| **VABS-II Communication** | 24 | 64.58 (12.17) | 34-89 | 23 | 58.7 (22.2) | 20-93 | 0.92 |
| **VABS-II Social Skills** | 24 | 63.92 (13.78) | 30-86 | 23 | 68.2 (10.7) | 41-92 | 0.09 |
| **VABS-II Daily Living** | 24 | 63.25 (24.5) | 20-90 | 23 | 71.6 (14.8) | 21-93 | 0.05 |

ADOS-2: Autism Diagnostic Observation Schedule – Second Edition; CS: Composite Score; CVP: Cognitive Verbal-Preverbal; EL: Expressive Language; FM: Fine Motor; GM: Gross Motor; GMDS-ER: Griffith Mental Developmental Scale - Extended Revised; GQ: Global Quotient; PEP: Psychoeducational Profile; RL: Receptive Language; RRB: Repetitive and Restricted Behaviors SA: Social Affect; VABS-II: Vineland Adaptive Behavior Scale – Second Edition; VMI: Visuo-motor Imitation.

**Supplementary Table S2.** CGI-I scores and ESDM response profiles of the 32 children enrolled in this study, followed by the T2 – T0 % difference in GMDS-ER subscales (n = 32) and ADOS-2 Total score (n = 24).

Improvement by > 25% is highlighted in bold and gray.

† GMDS-ER % difference not informative of treatment response, because GQ >70 already at intake.

|  | **CGI-I score** | **ESDM response profile** | **GMDS-ER (% diff)** | | | | | | **ADOS-2 total score (% diff)** |
| --- | --- | --- | --- | --- | --- | --- | --- | --- | --- |
| **Id. n.** |  |  | **Locomotor** | **Personal-Social** | **Language** | **Visuomotor Coordination** | **Performance** | **GQ** |  |
| 1 | 1 | Full Responder | **118.75** | -9.21 | **90.7** | -13.92 | 21.69 | 24.24 | n.a. |
| 2 | 1 | Full Responder | **44.26** | **41.56** | **61.9** | **61.76** | **59.74** | 52.05† | n.a. |
| 3 | 1 | Full Responder | -10.05 | 3.54 | 17.5 | 6.82 | -6.3 | 0.00† | **-33.33** |
| 4 | 1 | Full Responder | -10.39 | 14.29 | **89.29** | -10.98 | 6.49 | 7.81 | **-57.14** |
| 5 | 2 | Partial Responder | 14.81 | **32** | **53.13** | **61.76** | **158.82** | 62.86 | n.a. |
| 6 | 2 | Partial Responder | -31.45 | 10.58 | **81.55** | -17.5 | -14.38 | -8.16† | -7.69 |
| 7 | 2 | Partial Responder | -13.87 | **33.56** | **87.5** | **32.72** | 6.03 | 17.82† | **-31.25** |
| 8 | 2 | Partial Responder | **113.96** | **141.79** | -21.43 | **170.59** | **150** | **104.76** | **-36.67** |
| 9 | 2 | Partial Responder | 0 | 19.57 | 2.5 | **96.67** | **38.3** | **26.67** | **-40** |
| 10 | 2 | Partial Responder | **58.62** | **110.53** | **64.29** | **37.5** | 17.46 | **52** | **-50** |
| 11 | 2 | Partial Responder | **47.37** | **56** | **87.8** | 10 | 13.64 | **40.74** | **-33.33** |
| 12 | 2 | Partial Responder | **31.03** | 19.67 | 6.85 | 20.29 | -15.12 | 8.7 | -16.67 |
| 13 | 3 | Low Responder | 1.72 | 9.26 | -26.09 | 3.08 | 2.94 | 0 | 0 |
| 14 | 3 | Low Responder | 19.23 | -8.33 | **68.97** | **83.33** | **114.63** | **58.33** | n.a. |
| 15 | 3 | Low Responder | -20.51 | -48.08 | 9.76 | -28.85 | -30.16 | -25.86 | n.a. |
| 16 | 3 | Low Responder | 10.94 | 21.95 | -9.76 | 1.56 | -2.74 | 7.14 | n.a. |
| 17 | 3 | Low Responder | -20.83 | -13.16 | 5.36 | -13.64 | 25 | -4.88† | n.a. |
| 18 | 3 | Low Responder | -13.68 | **34.55** | -60.19 | -13.59 | -- | -20.00† | n.a. |
| 19 | 3 | Low Responder | -71.43 | -68.65 | -75 | -68.63 | -81.52 | -25.81 | 0 |
| 20 | 3 | Low Responder | -8.35 | -8.11 | -14.88 | -14.66 | -26.09 | -14.29† | 25 |
| 21 | 3 | Low Responder | -0.72 | -2.03 | -12.58 | -4.76 | 13.39 | -1.94 | -22.73 |
| 22 | 3 | Low Responder | 9.59 | 11.15 | -3.56 | 9.03 | -12.05 | 5.85 | 72.73 |
| 23 | 3 | Low Responder | **45.44** | 20.62 | **116.67** | **32.95** | 0.14 | **32.6** | 7.14 |
| 24 | 3 | Low Responder | 7.14 | 12.24 | -10.31 | 2.74 | **50** | 12.24 | 25 |
| 25 | 3 | Low Responder | 6.67 | 13.21 | -25.53 | 3.17 | **31.6** | 3.17 | -21.05 |
| 26 | 3 | Low Responder | -6.25 | -24.19 | -39.58 | -3.17 | -29.23 | -25 | 9.52 |
| 27 | 3 | Low Responder | -27.63 | **126.32** | -44.83 | 16.67 | -9.38 | 2.13 | 9.52 |
| 28 | 3 | Low Responder | **27.27** | 6.52 | -17.02 | 0 | 6.82 | 4.35 | -23.81 |
| 29 | 3 | Low Responder | **38.98** | **25.58** | -21.05 | 15.38 | 0 | 14 | 10 |
| 30 | 3 | Low Responder | **28.3** | -6.38 | **26.67** | -11.9 | -2.08 | -16.67 | -19.05 |
| 31 | 3 | Low Responder | -10.87 | -29.63 | -29.27 | -22.03 | -28.99 | -22.68 | -5.26 |
| 32 | 3 | Low Responder | **50** | 5.17 | 0 | -17.57 | 12.35 | 10.58 | -13.04 |
|  |  |  |  |  |  |  |  |  |  |

ADOS-2: Autism Diagnostic Observation Schedule – Second Edition; GMDS-ER: Griffiths Mental Developmental Scale - Extended Revised; GQ: Global Quotient. n.a.: not available.

**Supplementary Table S3.** Estimates, coefficients, statistics and p-values of non-significant Fisher’s Exact Test and logistic regression analyses of all variables assessed as possible predictors of response to ESDM treatment, including pretreatment ADOS-2, ADI-R, GMDS-ER, PEP-3 and VABS-II subscale scores, joint attention, imitation, eye contact, play skills and chronological age (see Methods).

|  |  |  |  |  |  |  | ***Overall goodness of fit*** | |
| --- | --- | --- | --- | --- | --- | --- | --- | --- |
|  | ***N*** | ***b*** | **SE B** | ***z*** | ***p*** | **OR (CI)** | ***χ²*** | ***p*** |
| **ADI-R A** | 25 | -0.05 | 0.08 | -0.66 | 0.50 | 0.95 (0.80-1.12) | 0.43 | 0.51 |
| **ADI-R B** | 25 | 0.00 | 0.09 | 0.04 | 0.96 | 1.00 (0.84-1.23) | 0.00 | 0.97 |
| **ADI-R D** | 24 | -0.01 | 0.45 | -0.02 | 0.98 | 0.99 (0.40-2.57) | 4e-04 | 0.98 |
| **ADOS-2 RRB** | 24 | -0.12 | 0.21 | -0.56 | 0.58 | 0.89 (0.55-1.30) | 0.34 | 0.56 |
| **ADOS-2 SA** | 24 | -0.17 | 0.12 | -1.45 | 0.15 | 0.85 (0.66-1.05) | 2.20 | 0.14 |
| **ADOS-2 Total** | 24 | -0.14 | 0.10 | -1.40 | 0.16 | 0.87 (0.70-1.04) | 2.20 | 0.14 |
| **Age at intake** | 32 | 0.08 | 0.09 | 0.98 | 0.33 | 1.08 (0.92-1.30) | 1.00 | 0.32 |
| **Eye Contact^†^** | 31 | --- | --- | --- | 0.07 | --- | --- | --- |
| **GMDS-ER GQ** | 32 | 0.02 | 0.02 | 0.99 | 0.32 | 1.02 (0.98-1.06) | 0.99 | 0.32 |
| **GMDS-ER Locomotor** | 32 | 0.01 | 0.01 | 0.38 | 0.70 | 1.00(0.97-1.03) | 0.14 | 0.71 |
| **GMDS-ER Language** | 32 | 0.02 | 0.02 | 0.87 | 0.39 | 1.02 (0.98-1.06) | 0.76 | 0.38 |
| **GMDS-ER Visuomotor Coordination** | 32 | 0.01 | 0.02 | 0.70 | 0.48 | 1.01 (0.98-1.05) | 0.50 | 0.48 |
| **GMDS-ER Performance** | 31 | 0.02 | 0.01 | 1.43 | 0.15 | 1.02 (0.99-1.05) | 2.11 | 0.15 |
| **Imitation^†^** | 29 | --- | --- | --- | 0.12 | --- | --- | --- |
| **PEP-3 Fine Motor** | 25 | 0.04 | 0.03 | 1.45 | 0.15 | 1.04 (0.98-1.09) | 2.22 | 0.13 |
| **PEP-3 Gross Motor** | 25 | 0.02 | 0.02 | 0.96 | 0.34 | 1.02 (0.98-1.07) | 0.92 | 0.34 |
| **Play^†^** | 31 | --- | --- | --- | 0.15 | --- | --- | --- |
| **VABS-II Daily Living** | 24 | 0.01 | 0.02 | 0.67 | 0.50 | 1.01 (0.98-1.05) | 0.47 | 0.49 |
| **VABS-II Composite Score** | 24 | 0.03 | 0.04 | 0.80 | 0.42 | 1.03 (0.96-1.11) | 0.67 | 0.41 |
| **VABS-II Motor Skills** | 24 | 0.11 | 0.06 | 1.79 | 0.07 | 1.12 (0.99-1.29) | 3.82 | 0.05 |
| **VABS-II Social Skills** | 24 | 0.00 | 0.04 | 0.07 | 0.94 | 1.00 (0.94-1.07) | 0.00 | 0.94 |

**^†^**Fisher’s exact test.

ADI-R: Autism Diagnostic Interview – Revised; ADOS-2: Autism Diagnostic Observation Schedule – Second Edition; GMDS-ER: Griffith Mental Developmental Scale – Extended Revised; GQ: Global Quotient; PEP: Psychoeducational Profile; RRB: Repetitive and Restricted Behaviors; SA: Social Affect; VABS-II: Vineland Adaptive Behavioral Scales – Second Edition.
